# Over-the-counter antibiotic sales in community and online pharmacies, China

**DOI:** 10.2471/BLT.19.242370

**Published:** 2020-04-21

**Authors:** Yanhong Gong, Nan Jiang, Zhenyuan Chen, Jing Wang, Jia Zhang, Jie Feng, Zuxun Lu, Xiaoxv Yin

**Affiliations:** a Department of Social Medicine and Health Management, School of Public Health, Tongji Medical College, Huazhong University of Science and Technology, Wuhan 430030, China.

## Abstract

**Objective:**

To determine the prevalence of and factors associated with dispensing antibiotics without a prescription in online and community pharmacies in China.

**Methods:**

We conducted a nationwide cross-sectional study of online and community pharmacies in 27 cities and counties in nine provinces in China (selected by multistage sampling) from July 2017 to December 2018. We assessed sale of antibiotics without a prescription and quality of pharmacy services through simulated clients who asked to buy specific antibiotics. We compared the prevalence of sales between online and community pharmacies, and between location and features of community pharmacies.

**Findings:**

Of 220 online and 675 community pharmacies, 174 (79.1%) and 586 (86.8%) sold antibiotics without a valid prescription, respectively. About half of the online pharmacies had a notice on their website about the illegality of selling prescription-only medicines without a prescription while none of the community pharmacists had. More online pharmacies without this notice dispensed antibiotics without a valid prescription (*P* < 0.001). Antibiotics’ sale without a prescription was significantly less prevalent in provincial capital cities (71.6%; 161/225) than prefectural-level cities (95.1%; 214/225) and counties (93.8%; 211/225; *P* < 0.001). Most pharmacy staff did not ask for important information from clients before dispensing the antibiotic or provide them with necessary information about the antibiotic.

**Conclusion:**

Given the high proportion of sales of prescription-only medicines without a prescription, there is a need to strengthen enforcement of regulations, improve public education on antibiotics, train pharmacy staff and consolidate public involvement in antibiotic stewardship in retail pharmacies in China.

## Résumé

**Objectif:**

Déterminer la fréquence à laquelle des antibiotiques sans ordonnance sont vendus dans les pharmacies communautaires et en ligne, ainsi que les facteurs qui y sont associés.

**Méthodes:**

Nous avons mené une étude transversale à l'échelle nationale auprès de pharmacies communautaires et en ligne, dans 27 villes et comtés de neuf provinces chinoises (sélectionnées selon un échantillonnage à plusieurs degrés), entre juillet 2017 et décembre 2018. Nous avons analysé la vente d'antibiotiques sans ordonnance et évalué la qualité des services pharmaceutiques en nous faisant passer pour des clients cherchant à acheter des antibiotiques spécifiques. Enfin, nous avons comparé la fréquence de vente entre pharmacies en ligne et pharmacies communautaires, de même que la localisation et les caractéristiques des pharmacies communautaires.

**Résultats:**

Sur les 220 pharmacies en ligne et 675 pharmacies communautaires observées, 174 (79,1%) et 586 (86,8%) respectivement ont vendu des antibiotiques sans ordonnance valable. Près de la moitié des pharmacies en ligne avaient publié un avertissement sur leur site précisant que la vente sans ordonnance de médicaments soumis à prescription était illégale; en revanche, aucune pharmacie communautaire ne mentionnait cette information. Les pharmacies en ligne vendant des antibiotiques sans ordonnance valable étaient souvent celles qui n'avaient pas publié cet avertissement (*P* < 0,001). La vente d'antibiotiques sans ordonnance était nettement moins fréquente dans les capitales provinciales (71,6%; 161/225) que dans les villes de niveau préfectoral (95,1%; 214/225) et les comtés (93,8%; 211/225; *P* < 0,001). La plupart des pharmaciens ne posaient pas aux clients les questions de base avant de leur fournir l'antibiotique, ou ne leur communiquaient pas les renseignements nécessaires à son propos.

**Conclusion:**

Étant donné la forte proportion de vente sans ordonnance de médicaments soumis à prescription, il est impératif de renforcer l'application de la réglementation, de mieux sensibiliser le public sur les antibiotiques, de former les pharmaciens et de promouvoir la participation de la population aux activités de gestion des antibiotiques dans les pharmacies de détail en Chine.

## Resumen

**Objetivo:**

Determinar la prevalencia de la distribución de los antibióticos sin receta en las farmacias en línea y comunitarias de China, así como los factores relacionados con esa distribución.

**Métodos:**

Se realizó un estudio transversal a nivel nacional de las farmacias en línea y comunitarias en 27 ciudades y condados de 9 provincias de China (seleccionadas mediante un muestreo de etapas múltiples) entre julio de 2017 y diciembre de 2018. Se evaluó la venta de antibióticos sin receta y la calidad de los servicios de las farmacias a través de clientes falsos que solicitaban comprar antibióticos específicos. Se comparó la prevalencia de las ventas entre las farmacias en línea y las comunitarias, y entre la ubicación y las características de las farmacias comunitarias.

**Resultados:**

De 220 farmacias en línea y 675 farmacias comunitarias, 174 (79,1 %) y 586 (86,8 %) vendieron antibióticos sin recetas válidas, respectivamente. Alrededor de la mitad de las farmacias en línea tenían un aviso en su sitio web sobre la ilegalidad de la venta sin la receta de los medicamentos que solo se venden con receta, mientras que ninguna de las farmacias comunitarias lo tenía. Más farmacias en línea sin este aviso suministraban antibióticos sin recetas válidas (P < 0,001). La venta de los antibióticos sin receta fue significativamente menos frecuente en las capitales de provincia (71,6 %; 161/225) que en las ciudades con nivel prefectural (95,1 %; 214/225) y en los condados (93,8 %; 211/225; P < 0,001). La mayoría del personal de las farmacias no pedía información importante a los clientes antes de vender los antibióticos ni les proporcionaba la información necesaria sobre su uso.

**Conclusión:**

Teniendo en cuenta el alto porcentaje de ventas sin la receta de los medicamentos sujetos a receta médica, es necesario reforzar la aplicación de las regulaciones, mejorar la educación pública sobre los antibióticos, capacitar al personal de las farmacias y consolidar la participación pública en la administración de los antibióticos en las farmacias minoristas de China.

## ملخص

الغرض تحديد مدى انتشار صرف المضادات الحيوية دون وصفة طبية في صيدليات الإنترنت وصيدليات المجتمع في الصين، والعوامل المرتبطة بذلك.

الطريقة قم بإجراء دراسة مستعرضة على مستوى الدولة للصيدليات عبر الإنترنت وصيدليات المجتمع في 27 مدينة ومقاطعة في تسعة أقاليم بالصين (تم اختيارها عن طريق أخذ عينات متعددة المراحل) من يوليو/تموز 2017 إلى ديسمبر/كانون أول 2018. وقمنا بتقييم مبيعات المضادات الحيوية دون وصفة طبية، وجودة خدمات الصيدلة من خلال عملاء محاكاة طلبوا شراء مضادات حيوية محددة. كذلك قمنا بمقارنة انتشار المبيعات بين الصيدليات عبر الإنترنت وصيدليات المجتمع، وبين موقع وميزات صيدليات المجتمع.

النتائج من بين 220 صيدلية عبر الإنترنت، و675 صيدلية بالمجتمع، قامت 174 صيدلية (بنسبة 79.1٪) و586 صيدلية (بنسبة 86.8٪) على التوالي، ببيع مضادات حيوية دون وصفة طبية صالحة. قرابة نصف الصيدليات عبر الإنترنت، وضعت إشعار على موقعها الإلكترونية بخصوص عدم شرعية بيع أدوية الوصفات طبية دون وصفة طبية، بينما لم يكن تضع أي من صيدليات المجتمع مثل هذا الإشعار. قامت المزيد من الصيدليات عبر الإنترنت، لا تضع هذا الإشعار، بصرف المضادات الحيوية دون وصفة طبية صالحة (نسبة الاحتمال أقل من 0.001). كان بيع المضادات الحيوية دون وصفة طبية أقل انتشاراً بشكل ملحوظ في عواصم الأقاليم (71.6٪؛ 161/225)، عنه في المدن على مستوى المحافظات (95.1٪؛ 214/225)، والمقاطعات (93.8٪؛ 211/225؛ نسبة الاحتمال أقل من < 0.001). لم يطلب معظم فرق العمل بالصيدليات معلومات هامة من العملاء قبل صرف المضاد الحيوي، أو تزويد العملاء بالمعلومات اللازمة عن المضاد الحيوي.

الاستنتاج نظراً للنسبة العالية لمبيعات أدوية الوصفة الطبية دون وصفة طبية، فإن هناك حاجة إلى دعم تطبيق اللوائح، وتحسين التوعية العامة بالمضادات الحيوية، وتدريب فرق العمل بالصيدليات، وتدعيم المشاركة العامة في الإشراف على المضادات الحيوية في صيدليات البيع بالتجزئة في الصين. ****

## 摘要

**目的:**

旨在确定中国网上药店和社区药店在没有处方的情况下配售抗生素的状况以及与之相关的因素。

**方法:**

从 2017 年 7 月至 2018 年 12 月，我们以中国的网上药店和 9个省的 27 个市县的社区药店（通过多阶段抽样选择）为研究对象，在全国范围内进行了现况调查。我们通过模拟客户在没有处方的情况下要求购买特定抗生素的方法，评估了抗生素的销售情况以及药店的服务质量。我们分别对比了网上药店和社区药店、不同地区和特征的社区药店无处方销售抗生素的比例。

**结果:**

在 220 家网上药店和 675 家社区药店中，分别有 174 家 (79.1%) 和 586 家 (86.8%) 药店在没有有效处方的情况下销售抗生素。大约一半的网上药店在其网站上发布了关于在没有处方的情况下销售处方药是违法的通知，而社区药店都没有。没有发布此类通知的网上药店更容易在没有有效处方的情况下配售抗生素 (P<0.001)。省会城市 (71.6%; 161/225) 在没有处方的情况下销售抗生素的比例明显低于地级市 (95.1%; 214/225) 县 (93.8%; 211/225; P < 0.001) 。大多数药房工作人员在配售抗生素之前并没有要求客户提供重要信息或向他们提供有关抗生素的必要信息。

**结论:**

鉴于在没有处方的情况下销售处方药的比例很高，因此有必要加强法规的执行力度、改进有关抗生素的公众教育、培训药店工作人员并且进一步鼓励公众参与中国零售药店的抗生素管控。

## Резюме

**Цель:**

Определить, насколько распространена и с какими факторами связана продажа антибиотиков без рецепта в местных и онлайн-аптеках Китая.

**Методы:**

Авторы провели национальное одномоментное поперечное углубленное исследование местных и онлайн-аптек в 27 городах и округах девяти провинций Китая (отобранных посредством многоступенчатой выборки) в период с июля 2017 года по декабрь 2018 года. Для оценки продаж антибиотиков без рецепта, а также качества обслуживания аптеками в целом исследователи представлялись покупателями и просили продать им определенные антибиотики. Авторы сравнили долю продаж в местных и онлайн-аптеках, а также провели сравнение по местонахождению и характеристикам местных аптек.

**Результаты:**

Из 220 онлайн-аптек и 675 местных аптек, включенных в исследование, 174 (79,1%) и 586 (86,8%) аптек соответственно продавали антибиотики без действительного рецепта. Примерно в половине онлайн-аптек на сайте имелось уведомление о том, что продажа рецептурных препаратов без рецепта противозаконна, тогда как в местных аптеках такого уведомления не было. Среди онлайн-аптек, отпускавших антибиотики без рецепта, превалировали аптеки, в которых отсутствовало уведомление (*P* < 0,001). Безрецептурная продажа антибиотиков значительно реже встречалась в городах-столицах провинций (71,6%; 161/225), чем в городах уровня префектур (95,1%; 214/225) и округов (93,8%; 211/225; *P* < 0,001). Персонал аптек по большей части не запрашивал у клиентов важную информацию перед отпуском антибиотиков и не сообщал им необходимые сведения об антибиотиках.

**Вывод:**

Учитывая высокую долю продаж рецептурных препаратов без рецепта, необходимо принять меры по обеспечению соблюдения нормативных актов, повысить осведомленность общественности относительно антибиотиков, обучать персонал аптек и вовлекать общественность в процесс надзора за отпуском антибиотиков в розничной аптечной сети Китая.

## Introduction

Antibiotic misuse and overuse and the antibiotic resistance these practices have caused are serious global public health problems.^1^^–^^4^ Pharmacies are one of the main sources of antibiotics worldwide;^4^^,^^5^ an average of 93% of people reported to have obtained their most recently used antibiotics from pharmacies.^2^ Although antibiotics are prescription-only medicines in many countries, they are frequently supplied without a prescription across the world. The overall global prevalence of dispensing antibiotics without a prescription in community pharmacies is 62.2%.^6^ Such antibiotic dispensing is particularly prevalent in Saudi Arabia (97.9%; 47/48 transactions), India (94.3%; 248/263 transactions), Indonesia (90.9%; 80/88 transactions) and Syrian Arab Republic (87.0%; 174/200 transactions).^7^^–^^10^

With the development of the internet, online sales of antibiotics have increased public access to these medicines, which is an additional challenge for antibiotic stewardship.^11^^,^^12^ Research has shown that antibiotics were freely available for purchase on the internet without a prescription for a buyer from Canada, the United Kingdom of Great Britain and Northern Ireland and the United States of America, which encourages self-medication and poor quality of care.^13^ Other research in the United Kingdom reported that 45.0% (9/20) of online pharmacies dispensed antibiotics without a prescription.^12^

China has a serious problem of antibiotic misuse and overuse, and retail pharmacies are the most common source of non-prescription antibiotics. Previous research has shown that about 70–84% of community pharmacies in China dispensed antibiotics without a prescription for adults with acute upper respiratory infections.^14^^–^^16^ The Chinese government has issued policies and regulations to improve the rational use of antibiotics in retail pharmacies, and a 2004 law stipulated that pharmacies are only permitted to dispense antibiotics with a prescription.^17^ In addition, retail pharmacies should have a separate counter for displaying antibiotics and a sign stating “prescription-only medicine” posted on the counter indicating that antibiotics are sold only with a prescription. In addition, pharmacies are required to post a notice that asks customers to report any illegal drug dispensing of prescription-only drugs in a pharmacy or on a website by calling a dedicated telephone number and a reward will be given once verified.^18^ Furthermore, the 12th five-year plan on drug safety in 2012 explicitly stated that by 2015, all community and hospital pharmacies should have licensed pharmacists on duty during business hours to supervise the dispensing of medicines and ensure their rational use.^19^ However, by the end of 2017, there were 453 738 community pharmacies but only 408 431 licensed pharmacists in China.^20^ As a result of the shortage of licensed pharmacists and the lack of specific training on antibiotics for pharmacists, improving the rational use of antibiotics in retail pharmacies is not easy.^21^

In 2018, the drug sales of community pharmacies amounted to 391.9 billion Chinese yuan (¥; equivalent to 59.2 billion US dollars; US$), which accounted for about one fifth of the total drug sales (¥ 1.71 trillion; US$ 258.3 billion) in China.^22^ At the same time, with the development of electronic commerce, internet sales of medicines have increased substantially. Total drug sales on the internet in China increased by 29%; from ¥ 7.7 billion (US$ 1.1 billion) in 2017 to ¥ 9.9 billion (US$ 1.5 billion) in 2018.^22^ A 2017 study on online drug-purchasing in China reported that 46.9% (183/390) of participants had bought medicines on the internet.^23^ In view of the considerable harm caused by antibiotic overuse and misuse, the large quantities of drugs sold in retail pharmacies and the rapid increase in online pharmacies, transparency about antibiotic dispensing in online and community pharmacies is important. Therefore, with the overarching objective of improving understanding of the current status of antibiotic sales in China, this study aimed to: (i) compare antibiotic dispensing in online and community pharmacies; (ii) explore factors associated with non-prescription antibiotic dispensing; and (iii) compare the quality of services of online and community pharmacies in terms of the information asked of and given to clients related to the sale of the antibiotic.

## Methods

We conducted a cross-sectional analysis of a nationally representative sample of online and community pharmacies in China from July 2017 to December 2018.

### Online pharmacies

The online pharmacy business in China operates in two ways. One way involves pharmaceutical enterprises that provide drug transaction services to customers directly through commercial websites. All such websites are displayed on the official website of the National Medical Products Administration (formerly the China Food and Drug Administration).^24^ The other way involves setting up online pharmacies on third-party trading platforms, with Taobao, Jingdong, Wechat, Amazon and Yihaodian being the five most popular platforms in China.

On 1 November 2018, we extracted all 693 domain names displayed by the National Medical Products Administration to purchase antibiotics through the websites. Of these domains, 364 could not be accessed because their domain names had expired, 109 only had medical devices or dietary supplements on sale, and the remaining 220 domains had drugs on sale. Of these 220 with drugs on sale, only 37 sold antibiotics.

For online pharmacies on third-party trading platforms (Amazon, Jingdong, Taobao, WeChat and Yihaodian), we searched for amoxicillin or cephalosporin on platforms directly to identify online pharmacies with antibiotics on sale. These two antibiotics are commonly distributed by retail pharmacies and are the most well-known antibiotics among the Chinese.^5^^,^^25^ Antibiotics were on sale at 88 (of 472) online pharmacies on WeChat, 72 (of 72) on Taobao, 23 (of 114) on Jingdong and none on Amazon or Yihaodian. We checked all 220 online pharmacies that had antibiotics on sale during November and December 2018.

### Community pharmacies

Community pharmacies were selected by multistage sampling across the mainland of China and investigated them from July 2017 to September 2018. We calculated the minimum number of pharmacies required within a surveyed province to estimate the proportions of non-prescription antibiotic dispensing in community pharmacies. We used the formula: *n* = z^2^*p*(1-*p*)/*d*^2^, where *n* is the sample size, *p* is the proportion of pharmacies dispensing antibiotics without a prescription, *z* is the normal deviation (1.96) and *d* is the margin of error (0.15*p*). Previous research on non-prescription antibiotic dispensing in community pharmacies in China had reported a prevalence of 67–84%.^14^^,^^26^ Therefore, we assumed that 70% of pharmacies dispensed antibiotics without a prescription (*P* = 0.7) and set the confidence interval (CI) at 95%. The minimum sample size calculated was 73 pharmacies for each province, which we increased to 75.

**Fig. 1:**
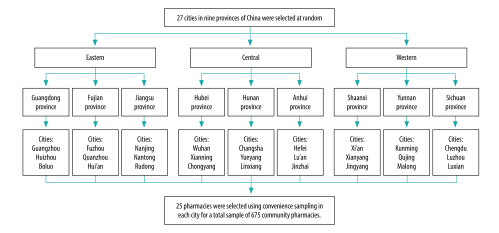
Flow diagram of sampling process of community pharmacies, China, 2018

The specific sampling process was as follows. First, based on the geographical area (according to the China health and family planning statistical yearbook),^27^ we divided China into three regions: eastern (11 provinces), central (8 provinces) and western (12 provinces). We selected three provinces from each region at random by drawing lots. Second, within each province, we included the provincial capital (the main city), and then randomly selected, by drawing lots, a prefecture-level city (smaller, less developed city than the provincial capital) and a county (rural area). Third, beginning at a general hospital in the central area of each city, the simulated clients visited a convenience sample of 25 community pharmacies, a total of 75 pharmacies in each province. In total, the simulated clients investigated 675 community pharmacies. The sampling process of community pharmacies is shown in Fig. 1.

### Simulated client

#### Online pharmacies

**Fig. 2:**
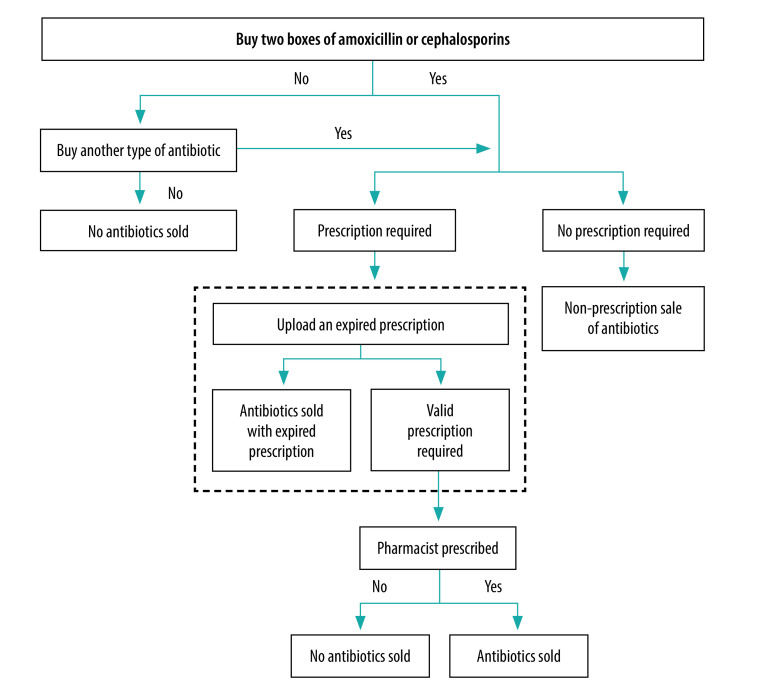
Flow diagram of the antibiotic purchasing process in online and community pharmacies, China, 2018

Note: Step shown in the dashed box was only carried out in the purchasing process for online pharmacies, not community pharmacies.

We recruited two postgraduate students in public health as the simulated clients.^28^ These students browsed online pharmacies and asked customer service personnel for two boxes of oral amoxicillin or cephalosporin. If neither medicine was on sale, they would ask for another type of antibiotic, such as azithromycin. If the online pharmacy stated that no antibiotics were for sale or that they did not dispense antibiotics without prescription, the investigation was concluded. When customer service personnel asked for a prescription before the sale of antibiotics, the simulated client uploaded an expired prescription that had been written 6 months earlier by a licensed doctor.^29^ If the pharmacy provided the antibiotics with this prescription, the investigation was concluded. If the pharmacy refused to dispense the antibiotic with this prescription, the simulated client asked the online pharmacists to prescribe a valid prescription for the antibiotic purchase. The purchase process is shown in Fig. 2. 

If asked about their reasons for purchasing antibiotics, the simulated client would reply, “I want to take a few boxes of antibiotics for a trip,” which implied there was no medical need to take antibiotics. Therefore, we defined customer service personnel’s agreement to sell antibiotics as illegal.

#### Community pharmacies

We recruited three undergraduate medical students to visit community pharmacies as simulated clients. The simulated client did not show any symptoms that required antibiotic use. If asked about their reasons for buying antibiotics, the simulated client gave the same reason as given to online pharmacies. The purchasing process at community pharmacies (Fig. 2) was similar to that in online pharmacies, except that no attempt was made to obtain antibiotics using an expired prescription because we did not have enough expired prescriptions.

#### Quality of services

After each investigation, the simulated client completed a standard questionnaire on whether or not the pharmacy had done the following: reminded the client about the illegal sale of prescription-only medicines; asked the client about their symptoms, drug allergies and history of taking the dispensed antibiotic; and dispensed the antibiotic. If the pharmacy dispensed the antibiotic, the simulated client also documented the prescription requirements, whether the pharmacist provided a prescription to dispense the antibiotic, and whether information was provided on the dosage of the antibiotic, duration of the course and possible adverse drug reactions. If the pharmacy did not dispense the antibiotic, the simulated client recorded the reasons for not dispensing them.

### Statistical analyses

We used SAS version 9.4 for Windows (SAS Institute Inc., Cary, NC, USA) for data analysis. We report descriptive statistical analyses as percentages. We used Fisher exact tests to compare the prevalence of sales of antibiotics without a prescription between online and community pharmacies, and between location and features of the community pharmacies. We did univariable and multivariable logistic regression analyses to assess the factors associated with community pharmacies dispensing antibiotics without a valid prescription. We considered differences to be statistically significant if *P* was less than 0.05.

### Ethics statement

The Ethics and Human Subject Committee of Tongji Medical College, Wuhan, China approved the research (no. 2014IEC(S078)). Given the minimal risk of the study to the pharmacies and to obtain realistic results, we did not disclose information about the investigation to the pharmacies. Since our objective was to ascertain whether antibiotics were being dispensed without a prescription, rather than reporting pharmacies for any illegal behaviour, all data were kept anonymous and confidential.

## Results

We found 878 online pharmacies that had drugs on sale, but only 220 (25.1%) sold antibiotics. We surveyed the 220 online pharmacies and 675 community pharmacies from nine provinces in China. Of the 220 online pharmacies, 111 (50.4%) had a notice for clients on their website that it was illegal to sell antibiotics without a prescription (Table 1), but none of the community pharmacists had such a notice. Of the 675 community pharmacies, 651 (96.4%) had a separate counter for dispensing antibiotics (Table 1).

**Table 1:** Antibiotic dispensing without a valid prescription in online and community pharmacies, by pharmacy characteristic, China, 2018

Characteristic	No. (%)							*P*^a^
	Total		Dispensed antibiotics					
			No		Yes			
					No prescription	Out-of-date prescription	Prescription provided by pharmacy	
**Online pharmacy**	220 (100.0)		46 (20.9)		118 (67.8)	44 (25.3)	12 (6.9)	NA
Notice posted on website that it is illegal to sell prescription-only drugs without a prescription								< 0.0001
No	109 (49.6)		17 (15.6)		84 (91.3)	8 (8.7)	0 (0.0)	
Yes	111 (50.4)		29 (26.1)		34 (41.5)	36 (43.9)	12 (14.6)	
**Community pharmacy**	675 (100.0)		89 (13.2)		568 (96.9)	NA	18 (3.1)	NA
Type of city								< 0.0001
Provincial capital city	225 (33.3)		64 (28.4)		143 (88.8)	NA	18 (11.2)	
Prefectural-level city	225 (33.3)		11 (4.9)		214 (100.0)	NA	0 (0.0)	
County	225 (33.3)		14 (6.2)		211 (100.0)	NA	0 (0.0)	
Geographic location								< 0.0001
East	225 (33.3)		38 (16.9)		187 (100.0)	NA	0 (0.0)	
Central	225 (33.3)		30 (13.3)		192 (98.5)	NA	3 (1.5)	
West	225 (33.3)		21 (9.3)		189 (92.6)	NA	15 (7.4)	
Separate counter for antibiotics								0.27
Yes	651 (96.4)		84 (12.9)		550 (97.0)	NA	17 (3.0)	
No	24 (3.6)		5 (20.8)		18 (94.7)	NA	1 (5.3)	
No. of pharmacy staff at work								0.21
1	148 (21.9)		16 (10.8)		130 (98.5)	NA	2 (1.5)	
2–3	390 (57.8)		49 (12.6)		331 (97.1)	NA	10 (2.9)	
≥ 4	137 (20.3)		24 (17.5)		107 (94.7)	NA	6 (5.3)	

NA: not applicable.

^a^ Fisher exact test.

In the survey of online pharmacies, we found three kinds of illegal sales of antibiotics: (i) sale of antibiotics without a prescription; (ii) sale of antibiotics with an expired prescription; and (iii) sale of antibiotics using a prescription provided by the pharmacy itself. Of the 220 online pharmacies that had antibiotics on sale, 174 (79.1%) agreed to dispense antibiotics without a valid prescription. Of the pharmacies that dispensed antibiotics, 118 (67.8%) sold these medicines without a prescription, 44 (25.3%) sold them after an expired prescription was uploaded and 12 (6.9%) dispensed them after the online pharmacy itself provided a prescription (Table 1). Online pharmacies without a notice about the illegal sale of prescription-only medicines were more likely to sell antibiotics illegally than pharmacies with this notice (*P* < 0.001; Table 1).

There were two types of illegal sale of antibiotics by community pharmacies: dispensing antibiotics without a prescription and dispensing antibiotics using a prescription provided by the pharmacy. Most of the community pharmacies (86.8%; 586/675) dispensed antibiotics without a valid prescription: 568 (96.9%) dispensed the antibiotics without any prescription and 18 (3.1%) dispensed them with a prescription provided by the pharmacy itself (Table 1). The prevalence of non-prescription sales of antibiotics in pharmacies in provincial capital cities (71.6%; 161/225) was significantly lower than that in prefectural-level cities (95.1%; 214/225) and counties (93.8%; 211/225; Table 1). Community pharmacy staff in prefectural-level cities (adjusted odds ratio, OR: 8.05; 95% CI: 4.08–15.89) or counties (adjusted OR: 6.09; 95% CI: 3.27–11.36) were significantly more likely to dispense antibiotics without a valid prescription than those in provincial capital cities. Furthermore, pharmacy staff in the western region were more likely to dispense antibiotics without a valid prescription than staff in the eastern region (adjusted OR: 2.35; 95% CI: 1.27–4.34; Table 2). Of the 89 community pharmacies that refused to dispense antibiotics, 84 did so because of the absence of a prescription. The other five considered it unnecessary for the client to have a reserve of antibiotics for a trip.

**Table 2:** Univariable and multivariable logistic regression of factors associated with dispensing antibiotics without a prescription in community pharmacies, China, 2018

Variable	OR (95% CI)	
	Unadjusted	Adjusted^a^
**City (Ref: provincial capital city)**		
Prefectural-level city	7.73 (3.95–15.14)	8.05 (4.08–15.89)
County	5.99 (3.24–11.07)	6.09 (3.27–11.36)
**Geographic location (Ref: east)**		
Central	1.32 (0.79–2.22)	1.50 (0.85–2.63)
West	1.97 (1.12–3.49)	2.35 (1.27–4.34)
**Counter for antibiotics (Ref: yes)**		
No	0.56 (0.21–1.55)	0.33 (0.11–1.01)
**Number of pharmacy staff at work (Ref: 1**)		
2–3	0.84 (0.46–1.54)	0.88 (0.46–1.66)
≥ 4	0.57 (0.29–1.13)	0.63 (0.30–1.31)

CI: confidence intervals; OR: odds ratios; Ref: reference category.

^a^ Adjusted for the other three variables.

Overall, 23 (10.4%) of the 220 staff from online pharmacies and 104 (15.4%) of the 675 community pharmacy staff enquired about allergies after the simulated clients asked to buy antibiotics. Of the 174 online pharmacies that agreed to dispense antibiotics, only one pharmacy explained the dosage, and two explained the possible adverse reactions. No online pharmacy explained the duration of the antibiotic course. For community pharmacies, 20.1% (118/586) and 5.3% (31/586) of pharmacy staff explained the dosage and duration of the course, respectively. No staff explained about adverse reactions (Table 3).

**Table 3:** Questions asked and information given by pharmacy staff to the simulated clients on antibiotics dispensed, China, 2018

Question or information	No. (%)	
	Online pharmacy	Community pharmacy
Asked about any drug allergies	23/220 (10.4)	104/675 (15.4)
Asked about history of taking the same antibiotic	29/220 (13.2)	175/675 (25.9)
Explained the dose of the antibiotic to take	1/174 (0.6)	118/586 (20.1)
Explained the duration of the antibiotic course	0/174 (0.0)	31/586 (5.3)
Explained the possible adverse reactions to the antibiotic	2/174 (1.1)	0/586 (0.0)

## Discussion

This study compares differences in antibiotic dispensing practices between online and community pharmacies using simulated clients. The simulated clients had no symptoms that required antibiotic use and therefore selling antibiotics to these customers was illegal.

The sale of prescription-only antibiotics without a valid prescription was common in both online and community pharmacies, particularly in community pharmacies in small cities and counties. Similar studies have shown that dispensing antibiotics without a prescription is prevalent in community pharmacies in China. The prevalence of dispensing antibiotics without a prescription in our study is similar to that reported in another Chinese study (83.6%; 925/1106),^16^ but higher than that reported in studies in 2017 (77.7%; 199/256) and 2019 (70.1%; 1690/2411).^14^^,^^15^ Nevertheless, these results all indicate that Chinese people can obtain antibiotics easily without a valid prescription and this problem requires urgent attention.

We found three types of illegal antibiotic dispensing in online pharmacies. While most of the pharmacies that sold the antibiotics did not require a prescription at all, 12 provided prescriptions and sold antibiotics after obtaining customers’ names and identity card numbers and 44 pharmacies accepted an expired prescription. Although the prevalence of these prescriptions is low, forged and invalid prescriptions increase the difficulty of enforcing the regulations on antibiotics. The government therefore needs to pay attention not only to whether antibiotics are sold with prescriptions, but also to the validity of these prescriptions through targeted management strategies.

We showed that compliance with the requirement to display a notice on the illegality of selling prescription-only medicine without a prescription was associated with fewer sales of antibiotics without a prescription in online pharmacies. However, despite the positive influence of this notice, online pharmacies still used invalid prescriptions to bypass the prescription requirement. In future, the notice should be strengthened to emphasize that prescriptions must be valid and legitimate. Although most community pharmacies had a separate counter for antibiotics, they nonetheless sold antibiotics without a prescription. Furthermore, none of the community pharmacies in the study displayed a public notice. These findings suggest that labelling medicines as prescription-only without empowering the public to actively monitor pharmacy practice does not reduce the sale of antibiotics without a prescription in community pharmacies.

In addition to dispensing antibiotics without a prescription, the quality of pharmacy services was poor, particularly in online pharmacies. Most pharmacy staff failed to ask for important information from the client before dispensing the medicine or to provide the client with needed information on the antibiotic. Lack of a trained pharmacist workforce to staff all retail pharmacies is an important reason for this problem.^20^ Pharmacy staff play an important role in advising the public on medicines, encouraging prudent use of antibiotics and reducing antimicrobial resistance. However, poor pharmacy practice is a common problem in developing countries.^6^^,^^30^ In 2012, the International Pharmaceutical Federation and the World Health Organization issued good pharmacy practice guidelines, which describes ways that pharmacists can improve access to health care, health promotion and the use of medicines for the clients they serve.^31^ Given that pharmacists are often the first contact that people have with the health-care system, some countries now provide national guidelines for pharmacists to tackle the sale of medicines without a prescription and increase pharmacy skills.^32^ National standards for good pharmacy practice have not yet been developed in China. However, our findings of antibiotic dispensing without a valid prescription and poor pharmacy practices, suggest that such standards are needed, especially in retail pharmacy. Pharmacy staff need training on what information they should ask from and give to the customer, and the importance of displaying notices in their pharmacies.

Although China has issued a series of policies and regulations to improve the rational use of antibiotics over the past decade, these regulations are often targeted at hospitals and overlook retail pharmacies. The easy access to antibiotics in retail pharmacies may encourage people to go to retail pharmacies instead of health-care institutions for antibiotics and suggests lax enforcement of the regulation on prescription-only medicines. This situation could pose a threat to antibiotic stewardship in health-care institutions introduced by the Chinese government in 2011. The Chinese government should recognize that the promotion of rational antibiotic use is complex and needs a systematic approach, and that decreasing antibiotic use only in health-care institutions is insufficient. Strengthening the enforcement of regulations, establishing a public supervision system and training pharmacy staff to improve the quality of their pharmaceutical services should be a priority of antibiotic stewardship for the Chinese government.

Although only 220 online pharmacies sell antibiotics, they provide services for people from all over the country. Therefore, monitoring and overseeing online pharmacies needs close attention. Due to restrictions on internet domain names, we did not collect data to determine whether online pharmacies sold antibiotics outside China. A previous study showed that British citizens could obtain antibiotics without a valid prescription from online pharmacies outside the United Kingdom.^9^ In the context of globalization, China should also introduce regulations to control the sale of antibiotics without a valid prescription to people living outside of China through online Chinese pharmacies.

Our study has some limitations. First, we evaluated requests for one product (antibiotics), which represents only a small number of pharmacy sales and may underestimate the prevalence of dispensing antibiotics without a prescription in online and community pharmacies. Second, our sample was recruited from nine provinces, but we did not apply probability proportionate to size sampling. This sampling resulted in an over-representation of pharmacies from prefecture-level cities and counties in the overall sample, which may overestimate the prevalence of antibiotics sale without a prescription in community pharmacies. Third, we selected community pharmacies based on convenience sampling, which could have led to selection bias and an underestimate of the prevalence of non-prescription antibiotic dispensing in community pharmacies.
